# VNFlow: integration of variational autoencoders and normalizing flows for novel molecular design

**DOI:** 10.1186/s13321-025-01104-2

**Published:** 2025-10-24

**Authors:** Jiří Hostaš, Mohammad S. Ghaemi, Hang Hu, Junan Lin, Anguang Hu, Hsu K. Ooi

**Affiliations:** 1 Digital Technologies Research Centre, National Research Council Canada, Toronto, ON Canada; 2 Suffield Research Centre, Defence Research and Development Canada, Medicine Hat, AB Canada

**Keywords:** Normalizing flows, Molecular design, Generative AI, Chemistry

## Abstract

Generative Artificial Intelligence is transforming the molecular discovery by enabling exploration of the vast, largely unexplored chemical space. However, current methods, including normalizing flows, struggle to balance the optimization of complex objectives and sampling speed, particularly when generating specific compound classes and more intricate scaffolds, such as aromatic rings. This work developed a generative model that efficiently samples novel molecules while optimizing their drug-likeness, ease of synthesis or chemical reactivity. To achieve this, we employed normalizing flows combined with variational autoencoders to generate samples which were evaluated for the Quantitative Estimate of Drug-likeness, the Synthetic Accessibility scores and, in case of organofluorine-phosphates, electronic density on the central phosphorus atom, approximated by Hirschfeld charges calculated with density functional theory. Our framework efficiently generated a diverse range of organofluorine-phosphates, demonstrating that combining normalizing flows directly with SELFIES or group-SELFIES can address key limitations in inverse molecular design, particularly when variational autoencoders cannot be applied due to a lack of available training data. Normalizing flows capture the chemical structures in a holistic way which paves the way towards targeted therapies that enable the optimization of complex molecular objectives.

**Supplementary Information:**

The online version contains supplementary material available at 10.1186/s13321-025-01104-2.

## Scientific contribution

To the best of our knowledge, this is the first report of integrating variational autoencoders with normalizing flows in a comprehensive molecular design workflow. It is also the first application of conditional normalizing flows to molecular design using string based molecular representation. Our method yielded novel molecules with performance metrics surpassing those in the 2.4 million-compound ChEMBL database, highlighting its potential for identifying promising drug candidates.

**Supplementary Information:**

The online version contains supplementary material available at 10.1186/s13321-025-01104-2.

## Introduction

The rapid growth in the amount of data, computing power, and advancements in artificial intelligence (AI) algorithms has led to the transformation of many scientific fields, including drug discovery [1]. This momentum was formally acknowledged in October 2024, when the Nobel Prizes in both Physics and Chemistry were awarded for the breakthroughs in training artificial neural networks and for solving the protein structure prediction challenge, respectively. However, studies estimate that the cost of developing a new drug remains in the range of a few billion dollars and can readily take more than 15 years to complete [2, 3]. The AI has the potential to accelerate rational design of new potential drug candidates, streamline clinical trial processes or democratize access to protein structure information which could significantly reduce the time and labor costs associated with drug development [4, 5]. In these and other applications, the early drug discovery methods continue to receive significant attention and efforts to address the challenges of exploring the vast, largely unknown, and yet to be thoroughly probed chemical space [6].

The initial stages of drug discovery, namely lead discovery and lead optimization were among the first implementations of AI models in chemistry. The generation of new drug leads without a deep prior knowledge is a focus of the so-called *de novo* molecular generation methods, which aim to improve molecular metrics such as drug-likeness, target specificity, and ADMET (Absorption, Distribution, Metabolism, Excretion, and Toxicity) [7]. Numerous AI models and databases have been developed to speed up the search through the chemical space which is mostly unexplored, large, discrete, and unstructured [8]. For a long time, the state-of-the-art approach has been to exhaustively scan and filter through fixed libraries or use discrete local search methods such as genetic algorithms [9]. This is highly impractical because it is not feasible in the near future to store or even search through the chemical space containing $10^{60}$ of all small molecules. Encouragingly, there have been methods which address some of these challenges by the conversion to continuous, data-driven, and machine-readable molecule representations and by generating new drug candidate molecules on demand. A good overview of the available methods can be found elsewhere, so let us point readers to the most popular machine learning solutions and highlight some of their differences [10].

First of the rapidly developing methods are generative adversarial networks (GANs). GANs operate through a competition between two models: one deep neural network generates new data with the same characteristics as the training set, while the second model, the discriminator, is trained to distinguish whether the generated data is part of the training set or not. A successful application for small molecules, MolGAN, was published by De Cao et al. where they focused on a direct generation of graph-structured molecular representations [11]. However, GANs are known to struggle with training instability and with generating larger and more complex molecules.

Since molecules can be viewed as sequences of strings and parentheses (representing atom types, bond types, and the starting and stopping points of the rings), the methods which proved their efficiency for the text generation, such as recurrent neural networks (RNNs), also found applications in the chemistry domain [12, 13]. The RNN models are known for an impressively high molecular validity rates reaching $98\%$ for a long-short term memory (LSTM) models developed by Bjerrum et al. or over $90\%$ for a gated recurrent unit (GRU) models, both examples of popular RNN architectures [14–16].

Gómez-Bombarelli et al. used one-hot encoded Simplified Molecular Input Line Entry System (SMILES) representation as a starting point for their variational autoencoder (VAE) [17]. The encoder part of the model was built using convolutional neural network layers while the decoder was the popular GRU model. A wide range of valid SMILES generation rates has been reported ranging between $\sim 75\%$ and $0.7\%$ depending on which part of the chemical space was sampled [18]. Although there exist other approaches with much higher decoding rates, such as the pure RNNs models with over $90\%$ decoding rates, a simple validity check can screen out invalid samples during the generation process and the latent representation can be leveraged for a straightforward visualization or as input for other models. Thus, the success rate of generating valid SMILES sequences is only one of the metrics in the model comparison and moreover, the goal of the molecular design is often an improvement across several competing and chemically relevant molecular properties.

Another family of deep generative models are normalizing flows and they are gaining popularity due to their applicability in a wide range of generative tasks [19]. These models provide competitive advantages over their GAN and VAE counterparts because they offer deterministic objective functions, efficient generative sampling and exact likelihood calculations. To the contrary, VAE models give only a lower-bound on log-likelihood while GANs do not provide any estimate of log-likelihood for its samples, which complicates their training and evaluation. Therefore, masked autoregressive flows, real non volume preserving flows and neural spline flows are some of the architectures designed to enable fast training or efficient sampling depending on the application and found their applications in image and graph generation applications [20, 21].

In the molecular design, one of the pioneering applications of normalizing flows is a flow-based graph generative model, MoFlow [22]. To generate a full molecular graphs in a one-shot manner, MoFlow starts by generating bonds (edges) with a Glow-based model, follows by atoms (nodes) attached using a graph conditional flow, and finalizes with posthoc validity correction checking for the valency constraints [23]. There are also hierarchical models, such as MolGrow, which generates molecular graphs iteratively via generating molecular graphs from a single-node graph by recursively splitting every node into two [24]. Chemical rules are also incorporated in the model called, GraphAF, which at the time outperformed other VAE based methods on molecular optimization tasks [25]. In the domain of 3D graphs, conditional normalizing flows were also used for a more complex task of generating ligand molecules capable of binding to a target receptor [26, 27]. They have been tested on large CrossDocked2020 set of over 22 million poses of ligands docked into receptors gathered from the Protein Data Bank [28].

Another application of normalizing flows for rapid molecular generation of small molecules was published by Frey et al. in 2022 [29]. Its simple architecture delivered an impressive efficiency in generating thousands of molecules per second, however, it was not optimized to address the molecular optimization of larger molecules or to systematically improve the studied molecular metrics. Instead, the authors lean on post-hoc filters and multi-objective optimization through high-throughput virtual screening.

Despite the improvements of the state-of-the-art normalizing flows architectures, they tend to be restrictive which leads to excessively large models and a high number of model parametres which hinders their training. In image generation, it has been shown that this can be partially addressed by VAE encoding through feature reduction [20, 30, 31]. In the molecular design domain, normalizing flows might improve valid SMILES generation rates of pseudo-randomly chosen points in the latent space of other models or help the VAE decoder to sample from the part of the space with novel molecules that have desired properties.

This work presents several novel applications of normalizing flows for molecular discovery. Firstly, we combine the normalizing flows model with VAE chemical encoding to improve sampling of new molecules and to reduce the number of molecular features and parameters needed to train our normalizing flows. The feature reduction increases sampling and training efficiency, which opens the door for testing a larger variety of normalizing flows. Secondly, we test the conditional normalizing flows for molecular design using string based molecular representation for the first time. Finally, we showcase an iterative design workflow in a low data regime where we generate organofluorine-phosphate molecules using normalizing flows. For the generated organofluorine-phosphate molecules, we optimize the relative atomic coordinates of their atoms and calculate their atomic charges with density functional theory (DFT).

## Methods

### Molecular representations, datasets and chemical metrics

The choice of input representation plays a key a role in the generative design and it is an ongoing area of research [32]. Here, we start with the most popular way to represent molecules as 1D SMILES (Simplified Molecular Input Line Entry System) strings which are sequences of ASCII characters that use a depth-first graph traversal [13]. This sequence of characters is converted into a one-hot-encoded vector, a representation eliminating ordinality (inherent order present in categorical representation) and enabling a straightforward application of various deep neural network architectures (RNN, VAE, GAN etc.) popular in natural language processing. Despite the popularity of SMILES, the methods built using this representation are prone to generating invalid SMILES strings that do not adhere to the rather strict SMILES notation.

There have been attempts to address these issues caused by the complex SMILES grammar. Krenn et al. introduced a new string representation called SELFIES (Self-Referencing Embedded Strings) which are completely robust while being less efficient for certain tasks [33]. Typically, the length of a SELFIES string is noticeably larger compared to SMILES which can inconvenience the applications of normalizing flow-based models. This has been partially addressed by group-SELFIES representation where a single SELFIES string can define a group of atoms chosen a priori [34]. For our application, we analyzed ZINC250k database using RDKit automatic fragmentation algorithms and identified 10 fragments with aromatic ring which were used to enrich the molecular generation of our tested models [35].

For example, the smallest molecular fragment in our dictionary (see the second molecule in Fig 12) is pyrrole. It can be written as “c1cc[nH]c1” using canonical SMILES or “[C][C][=C][NH1][C][=Ring1][Branch1]” using SELFIES representation. Please notice the increased length of the SELFIES string which is a typical consequence of using this fully robust and partially redundant representation. Finally, in group-SELFIES, we defined pyrrole group as C1=C(*1)C(*1)=C(*1)N1*1 in our dictionary file where *1 indicates location of an attachment site. This fragment can be applied in definition of the molecule as [:pyrrole] for a simple pyrrole or as part of a larger molecule, e.g. 2-methylpyrrole, as [1:pyrrole][C].

Most of the publicly available datasets store molecules as SMILES (ChEMBL, ZINC250K, QM9 etc.) [8, 35–37]. We trained several generative models on ChEMBL dataset of curated bioactive molecules [8]. We used a moderate-size dataset of 50,000 random ChEMBL22 structures (see analysis of QED and SA score in Table 1) for several initial tests and the latest (ChEMBL35) version of the entire dataset with $\sim ~2,400,000$ structures for further comparisons.

To assess the usefulness of the generated molecules, we evaluated the following metrics, which generally give an indication of which generated molecules might be interesting to study further: the quantitative estimate of drug-likeness (QED), Synthetic Accessibility (SA) score, Morgan fingerprint, the number of heavy atoms or aromatic rings; all of which were calculated using the RDKit package [38]. The QED and SA scores range from 0 to 1 and 1–10, respectively, where higher values indicate greater drug-likeness and greater synthetic difficulty. Consequently, it is desirable to generate molecules with high QED values and low SA scores. Further details on these metrics can be found in the RDKit documentation [38].

Although the molecules reside in 3D space and molecule’s conformations may be important for predictions of some properties such as binding strength to a specific receptor [39], most generative modelling methods are being developed primarily for the use with simpler graph and 1 d representations, especially in the case of modelling of large and flexible molecules. Typically, the bond length and 3D information are discarded, as is the case with our models. However, information about the energetically stable conformation can be recovered by energy minimization with quantum mechanical methods or empirical force fields. We have done this using density functional theory (DFT) in the case of organofluorine-phosphates (see Sects. 2.2 and .3.2).

### Density functional theory

Density Functional theory (DFT) has been applied in many applications from the description of electronic structure in materials science, protein-protein interactions, to drug-receptor interactions in drug discovery [40–42]. Here, DFT was used to test the stability and to probe the electronic-structure properties of the generated organo-phosphate molecules.

The geometry optimization, analytical harmonic vibrational frequencies and atomic Hirschfeld charges were calculated using Orca 5.0.4 software [43]. There are numerous ways and formulations which one can choose when applying DFT and we used B3LYP-D4 method with a medium-size (def-TZVP) basis set as a compromise between speed and accuracy [44–46]. The starting geometries were generated using RDKit [38] package.

### Variational autoencoders

**Fig. 1:**
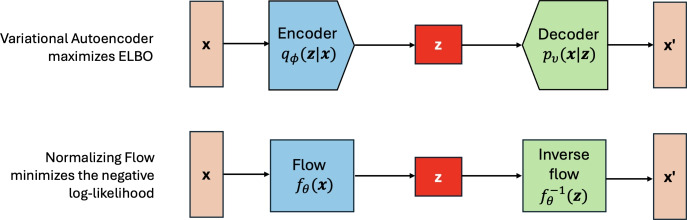
Comparison of the training of a variational autoencoder and normalizing flow models

Variational autoencoders (VAEs) are one of the most succesful and popular generative models in which the encoder - decoder pair is used to establish a discrete data mapping of ${\textbf {x}}$ (e.g. one-hot-encoded SMILES, SELFIES or group-SELFIES) to and from a latent continuous representation (${\textbf {z}}$, see the top panel of the Fig. 1). The encoder learns the underlying lower-dimensional distribution of features creating the data while the decoder generates new samples from this learned distribution. Alternatively, VAE can be understood as a collection of two coupled but independently parameterized models trained using stochastic gradient descent and maximizing the Evidence Lower Bound (ELBO) [47]. The ELBO consists of two main components: the reconstruction term ensuring that the latent space follows the original distribution and captures enough information to accurately reconstruct the input; and the regularization term (Kullback–Leibler divergence), which nudges the latent distribution to stay close to a predefined prior, typically a Gaussian distribution. A good introduction and discussion about the underlying model assumptions (e.g. about the latent spaces being close to normal) can be found elsewhere [48].

Our VAE models were derived from the architecture introduced by Gómez-Bombarelli et al. which was in turn based on the previous applications for English texts [17, 49]. Their architecture consists of three 1D convolution layers (having filter sizes of 9, 9, and 10 with the respective kernel sizes of 9, 9, and 11) and a fully connected linear layer. The latent space has 292 dimensions and the decoder uses three stacked layers of gated recurrent unit (GRU) networks each with a hidden dimension of 501 [50]. We used python package *pytorch* to implement it [51]. An example of our model convergence with respect to training and validation errors is depicted in the Fig. 15, while the performed hyperparameter search for the encoder architecture trained on SELFIES is in the Table 3. Several models were trained on 50,000 randomly sampled molecules (using 80:20 training/validation split) from the ChEMBL22 data set and 2.4 million molecules from the ChEMBL35 data set were converted into one-hot-encoded SMILES and SELFIES representations. These VAE models were used as our baseline models for generating novel molecules via decoding random vectors and also as a dimension reduction tools of the feature vectors for the normalizing flows.

### Normalizing flows

While most machine learning and AI tasks can be understood as learning the distributions of training data, only a minor subset of methods can, in principle, construct these distributions explicitly. Normalizing flows take a simple multivariate normal distribution (with the diagonal Gaussian density being the most popular choice) and apply a sequence of differentiable and invertible functions, also referred to as bijections or mappings:

1 $$\begin{aligned} f^{-1}_{\theta }=f_{\theta _{1}}^{-1} \circ f_{\theta _{2}}^{-1} \circ \dots \circ f_{\theta _{K}}^{-1} \end{aligned}$$

that are used to map it to the original, often complex, data distribution. These bijections have to be easy to compute and invert. Also, the determinant of their Jacobian should be easy to calculate, so one can compute the exact log-likelihood for each data point *x* by repeatedly applying the rule for change of variables as follows:

2 $$\begin{aligned} \log p_{\theta }(x) = \log p_{\iota }(z) + \sum _{i=1}^K\log \Big |\det \frac{\partial f_{\theta _{i}}^{-1}(x_i)}{\partial x_i}\Big | , \end{aligned}$$

where $p_{\theta }(x)$ is the data distribution, $p_{\iota }(z)$ is the known diagonal Gaussian distribution and $\Big | \det \frac{\partial f_{\theta _{i}}^{-1}(x_i)}{\partial x_i} \Big |$ is the determinant of the Jacobian matrix of the function mappings $f_{\theta _{i}}^{-1}(x_i)$. The mappings are typically implemented in a form of invertible deep neural networks and they can be trained by minimizing Kullback–Leibler divergence via minimizing the negative log-likelihood. One of the main limitations of normalizing flows is that they can be hard to train and the invertability criteria can limit the expressivity of the used transformations.

Several popular types of non-linear flows are derived from the coupling flow framework which repeatably splits the input into parts, where the first part stays the same while the second part undergoes a scale-and-shift (affine) transformation. Affine flows are defined by shifting the input by a bias value $\mu$ and scaled by a parameter $\sigma$, where $\mu$ and $\sigma$ are functions of the first part of the unchanged input and implemented as deep neural networks. The sums and multiplications are performed element-wise. The first type of flow tested here, based on the coupling of several of these affine transformations, is called Real Non Volume Preserving flow (Real NVP) and it is implemented in the python package *glasflow* [52]. Batch normalization was found to help with training models with a large number of coupling layers [52]. Since a subset of the input dimensions stay unmodified in each affine coupling layer, reverse or random permutations are applied (see more technical details in the *glasflow* documentation).

Affine flows have also been used within the autoregressive framework, which models data sequentially such that each dimension depends solely on the previously calculated dimensions. This has implications for sampling efficiency, as the sequential nature of the workflow prevents parallelization. In 2017, Masked Affine Autoregressive Flow (MAF) were introduced by Papamakarios et al. who introduced checkerboard masking which was tested it in its original *nflows* implementation [20, 53].

In 2019, the monotonic rational quadratic splines were introduced to enhance the flexibility of both coupling and autoregressive transforms while keeping the invertibility of the transforms [54]. We have used the implementation available in the package *normflows*, where the autoregressive rational quadratic neural spline transform is combined with lower-upper (LU) linear permute transformation [55]. For a comprehensive and more formal overview of normalizing flows, we refer readers to several in-depth review articles on the subject [56, 57].

**Fig. 2:**
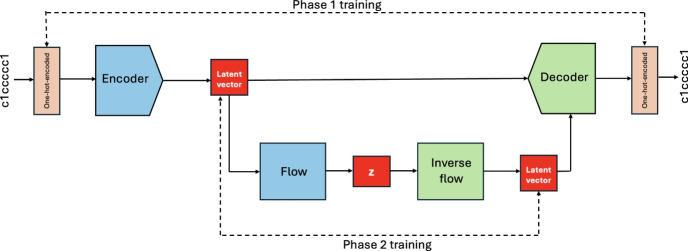
The integration of variational autoencoder and normalizing flow models. First, the variational autoencoder model was trained on the entire database (50,000 or 2.4 million examples) by maximizing the ELBO loss function. Subsequently, the normalizing flow model was trained on a subset of this database (the latent vectors of the SMILES strings with the top $1\%$ or, in case of the larger training dataset, the top $0.1\%$ QED score) by minimizing the negative log-likelihood

To generate novel molecules, we begin by sampling a random vector from a multivariate diagonal Gaussian distribution (${\textbf {z}}$ at the bottom of Fig. 2). This vector is then passed through the inverse flow, which consists of a previously trained sequence of bijective and differentiable functions. The output is decoded using the VAE decoder and the SMILES, SELFIES or group-SELFIES string is obtained by inverse transformation of the one-hot-encoded vector. Finally, the molecular string is validated and the desired properties of the novel molecules calculated.

Papamakarios et al. showed that incorporating additional information provided by labels can benefit the generative models [20]. We used QED scores or atomic charges as labels, allowing the normalizing flows to learn the conditional probability distributions, effectively doubling the number of models tested.

Our two phase training procedure is shown in the Fig. 2. All the components of the underlying VAE are fixed in the normalizing flow (second) training phase. It is possible to optimize the entire model, both VAE and normalizing flow, jointly in one phase, however, it was previously reported that it results in a poorer performance [30].

When there is a lack of training data for molecules with a specific desired functional group (e.g. organofluorine-phosphates, see Sect. 3.2), it is not possible to train a specialized VAE autoencoder encoding. Instead, we use SELFIES or group-SELFIES representations directly which proved to be more robust than using SMILES for a direct generation [33]. We apply a uniform noise in the range between 0 and 1 to dequantize the one-hot-encoded molecular vectors since the normalizing flows do not perform well with unprocessed binary data or discrete distributions [29].

Unless stated otherwise, we keep the number of coupling layers (4) and hidden features (32) fixed. The models are trained with the Adam optimizer, a batch size of 100, a step size between $10^{-3}$ and $10^{-5}$. Each model is trained using early stopping until no improvement is observed for 1000 epochs. We train normalizing flows on distributions of all available molecular samples or only on the target distribution of molecules (such as molecules with the top 0.1 or $1\%$ QED score, see Sect. 3.1) when stated. The train/test split was kept 80/20. In the case of the tests without VAE encoding and, therefore, the longer input lengths for normalizing flows, we used higher number of coupling layers (8) and hidden features (128).

### Computational resources and software

We performed all the simulations on a single machine with an Intel Xeon Silver 4214 2.20GHz CPU and a single NVIDIA GP107GL Quadro P1000 GPU with 4GB GDDR5 on-board memory. We used following python packages: *nflows* [53], *glasflow* [52], and *normflows* [55], *sci-kit learn* [58], *pytorch* [51], *RDKit* [38], *pandas* [59], and *numpy* [60]. The code will be made available upon publication.

**Table 1:** Molecular metrics for sets of molecules sampled by the VAE decoder, compared with samples from the ChEMBL database

Sampling method	Condi-	Novel, valid.	QED	SA score	Heavy atoms
	tioned	unique [$\%$]	mean/max	mean	mean
VAE trained on: SMILES (50,000 examples)					
Real NVP	No	1.0$\%$	**0.73**/0.9467	2.61	22
	Yes	1.1$\%$	**0.73**/**0.9478**	2.64	22
Masked Affine Autoregressive	No	1.1$\%$	0.70/0.9468	2.67	19
	Yes	0.9$\%$	0.72/0.9472	**2.49**	22
Autoreg. Rat. Quadr. Spline	No	0.9$\%$	0.46/0.9466	3.95	18

VAE trained on: SELFIES (50,000 examples)					
Real NVP	No	92.0$\%$	0.55/0.9468	4.14	17
	Yes	92.5$\%$	0.54/0.9470	4.18	17
Masked Affine Autoregressive	No	90.4$\%$	0.55/0.9475	4.08	17
	Yes	91.5$\%$	0.54/0.9473	4.13	17
Autoreg. Rat. Quadr. Spline	No	88.6$\%$	0.46/0.9468	4.51	20
Random$^1$		86.1$\%$	0.37/0.9450	4.85	19

VAE trained on: SMILES (2.4M examples)					
Real NVP	No	0.11$\%$	0.64/0.9077	5.14	24
	Yes	0.15$\%$	0.64/0.9276	5.01	24
Masked Affine Autoregressive	No	0.16$\%$	0.60/0.9302	5.03	26
Autoreg. Rat. Quadr. Spline	No	0.5$\%$	0.22/0.9058	4.91	48
Random$^1$		2.72$\%$	0.13/0.6881	5.22	68

ChEMBL22 (50,000 random samples)			0.76/0.9481	2.44	20
ChEMBL35 (2.4M samples)			0.56/0.9484	2.93	28

$^1$Random refers to random latent vectors decoded by the VAE

The best QED and SA scores among the normalizing flow models are highlighted in bold. A total of 100,000 samples were generated by each method

## Results and discussion

### Normalizing flows combined with variational autoencoder for the design of novel bio-active molecules

A major challenge with normalizing flows is their generally low expressibility, which limits their applicability to high-dimensional inputs. We addressed this issue through feature reduction in the molecular representation by using a VAE. It was trained in two phases: the VAE model was trained first on 50,000 random samples from the ChEMBL22 database, followed by the training of normalizing flows, which was performed only on the structures with the highest $1\%$ QED score. This approach improves the efficiency of training normalizing flows by reducing both the number of features and the number of samples on which they are trained.

**Fig. 3:**
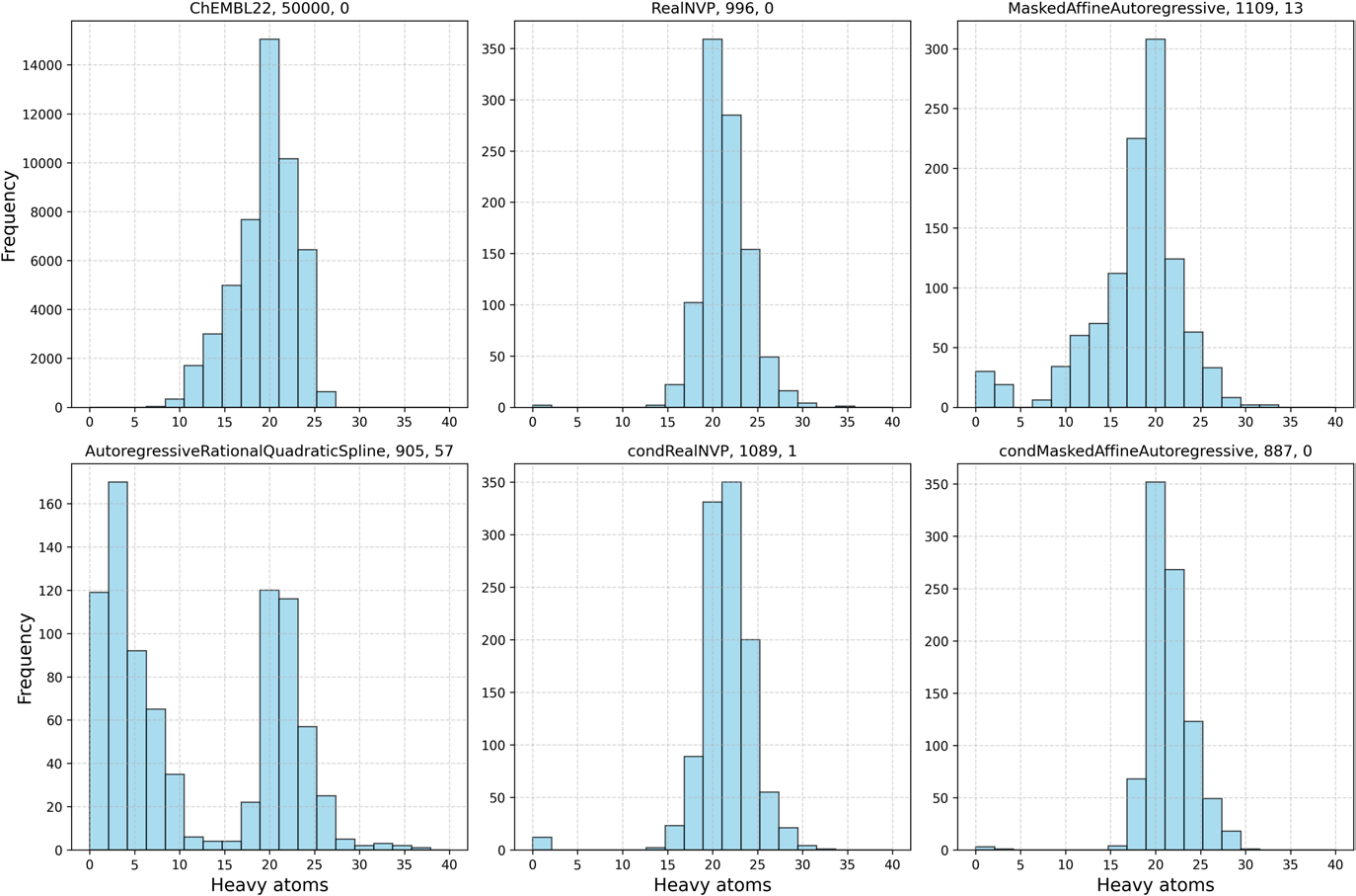
Histograms of the number of heavy atoms for the starting dataset (50,000 molecules from ChEMBL22 dataset, top left panel) compared with the histograms of the molecules generated by different normalizing flows (from the left middle to the bottom right: Real NVP, masked affine autoregressive, autoregressive rational quadratic spline, conditional Real NVP, and conditional masked affine autoregressive flows). Please note that the distributions are plotted only in the range from 0 to 40. The numbers on top of the graphs stand for the total number of structures in the set and the number of structures with more than 40 heavy atoms, respectively

**Fig. 4:**
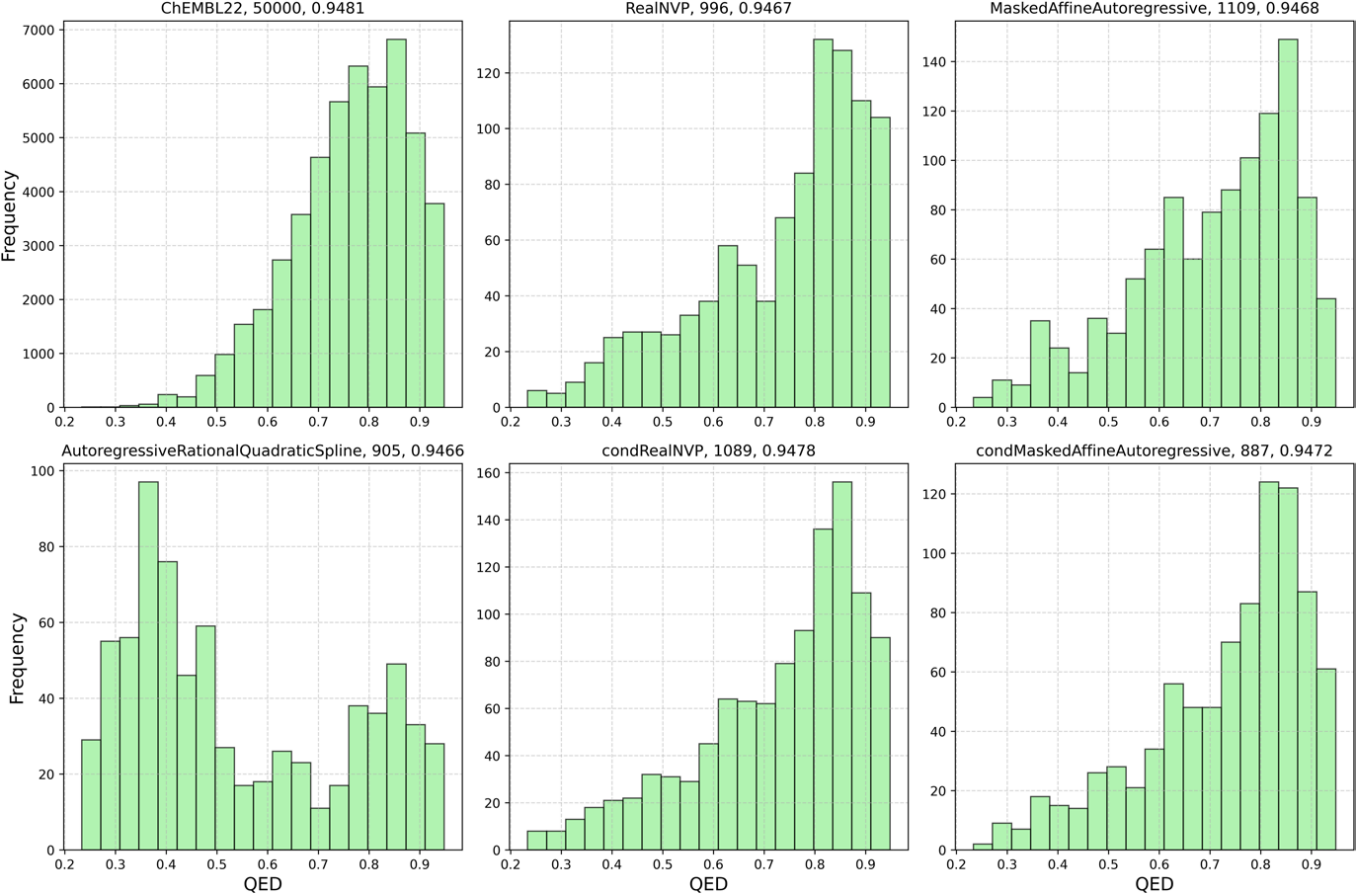
Histograms of the QED score calculated for the starting dataset (50,000 structures from ChEMBL22 dataset) compared with the histograms of the molecules generated by different normalizing flows (from the left middle to the bottom right: Real NVP, masked affine autoregressive, autoregressive rational quadratic spline, conditional Real NVP, and conditional masked affine autoregressive flows). The numbers on top of the graphs stand for the total number of structures plotted and the maximum QED value in the set, respectively

Essentially, our workflow aims to enhance the sampling efficiency of the VAE generative model by generating promising latent vectors using normalizing flows. To this end, we test several popular normalizing flows. The analysis of the training dataset is presented in Figs. 3, 4, 14, and Table 1. Most molecules contain between 15 and 25 heavy atoms, have QED scores ranging from 0.6 to 0.9, logP values between 0 and 5, and feature 1 to 3 aromatic rings.

Let us begin the analysis by comparing the performance of our VAE models with those of trained previously by Bombarelli et al. and Kusner et al. [17, 18] Table 1 shows that our VAE, using one-hot-encoded SMILES, achieved a sample validity rate of 0.8%, similar to the rates reported in previous studies. Higher rates were observed when interpolating between training data points or the sampled points are closer to the training data. Additionally, the more robust molecular representation (such as SELFIES, as shown in panel 2 of Table 1) resulted in a significantly higher validity rate of nearly $90\%$. However, in case of SELFIES, we observed a general decline in the quality of the samples documented by lowered QED scores and increased SA scores. This is because a SELFIES string gets truncated when a part of the SELFIES sequence or a single token does not result in a valid part of a molecule. On the other hand, SMILES string would simply be discarded. As a result, the average length of a SELFIES string is shorter, and the molecules are smaller, as evidenced by the lower mean number of heavy atoms (in our case ranging from 18 to 22 for samples generated with SMILES to 17–20 for SELFIES). Furthermore, the low validity rate is generally not a major issue, as filters and validity checks can quickly discard invalid SMILES strings. Thus, generating invalid strings can actually be beneficial for model development [61]. We trained the VAE model also on the entire ChEMBL35 dataset, containing 2.4 million SMILES. Notably, we observed an increase in the number of heavy atoms, resulting in molecules with a high count of heavy atoms, unremarkable QED scores, and exceptionally low validity rates. Further hyperparameter tuning may be required to effectively utilize a dataset of this size and diversity, however, this is beyond the scope of the current work.

Next, we focus on the results of the model trained on 50,000 SMILES examples, along with the overall performance of the normalizing flow architectures trained to generate VAE latent vectors. The corresponding results are summarized in Table 1. It is evident that the use of normalizing flows offers a substantial improvement over random sampling of the VAE latent space. While the increase in the percentage of valid and unique samples is relatively modest, the mean QED score is more than doubled, the maximum QED score achieved by any of the tested flows exceeds that obtained via random sampling, and the mean SA score is improved by approximately 40%. Figure 3 shows that all models, except for the autoregressive rational quadratic splines, generated molecules of similar size to those in the training dataset, with the mean number of heavy atoms around 20. In fact, all normalizing flow models, except for the autoregressive rational quadratic spline flow, generated molecules with distributions of QED scores similar to those in the training dataset (see the kernel density estimation of QED score in Fig. 11). Figure 4 also shows that the training dataset has a higher relative proportion of QED scores around the median value, compared to the normalizing flows, which exhibit extended tails in their QED score distributions, both in the lower and higher end. This is generally desired because we are typically looking for outliers with extremely high QED scores.

Clearly, Real NVP normalizing flow outperformed the masked affine autoregressive flow, and the same holds for their conditioned versions. The respective distributions are in the Fig. 4. Real NVP achieved also the highest number of structures with the high QED scores (the bin furthest to the right), which, even at a relative scale ($10\%$ of the total sampled structures), surpassed the $7\%$ of the ChEMBL training dataset. Additionally, among all the normalizing flows tested, Real NVP generated the molecule with the highest mean and maximum QED values, as shown in Table 1.

The autoregressive rational quadratic spline flows delivered the lowest average QED and the least promissing results (see Table 1). This can also be easily documented by the maximum of distribution of number of heavy atoms being at only 5 heavy atoms and with the largest number of samples with more than 40 heavy atoms (57 samples, see Fig. 3). This means that the autoregressive rational quadratic spline flows generated both, a large number of very short sequences and also excessively long strings. Notice that this behaviour is not apparent when only the averaged metrics in the Table 1 are considered. Furthermore, autoregressive rational spline flows were nearly two orders of magnitude slower producing only 3 molecules per second when compared to other normalizing flows. As a result, we do not recommend this type of flow for applications similar to those studied in this work.

Finally, we can conclude that the Real NVP bijections showed a great promise for sampling the latent space. Conditioning the normalizing flows on the QED score resulted in only a minor improvement for the masked affine autoregressive flow, while no clear improvement was observed for the conditional Real NVP flows. Thus, it remains unclear how to optimally leverage conditional normalizing flows to achieve an overall improvement. Normalizing flows when combined with a trained VAE decoder can play a role of samplers of distributions very close to the one that they were trained on. The behavior of different normalizing flows is obviously very empirical here, as it is not clear what is the optimal size of the training dataset and how tightly the normalizing flows should try to approximate the trained distributions in various applications. More tests and development will need to be done to explore the combinations of molecular representations and flows.

Next, we will address whether these methods can be applied for exploration outside the training domain. We will begin by using the model trained on 50,000 samples from the ChEMBL22 database and evaluate how many samples are needed to improve metrics such as QED scores.

#### Search for bio-active molecules with properties outside of the distribution of the training dataset

In the previous section 3.1, we demonstrated that normalizing flows can effectively model the distributions of the training dataset. However, we did not observe any improvement in the metrics of the generated molecules relative to those in the training dataset. This outcome was expected, given that the generation process started with a training dataset of 50,000 molecules, while the number of novel, valid, and unique molecules was limited to around 1,000 per normalizing flow during these initial experiments with one-hot-encoded SMILES representation.

Therefore, we continued generating molecules with the Real NVP normalizing flow combined with our VAE model. Among the newly generated samples, $0.98\%$ were valid and $0.75\%$ were unique. The vast majority of these samples were novel, with only a small fraction (1108 samples out of the first 250,000) overlapping with the original dataset. Furthermore, only 21 of these samples were re-invented and found to match entries in the considerably larger ChEMBL35 dataset, which contains 2.4 million molecules. These overlapping samples were excluded from further analysis, as they were not considered novel. Figure 17 shows the visualization of 250,000 generated molecules sampled across chemical space.

**Fig. 5:**
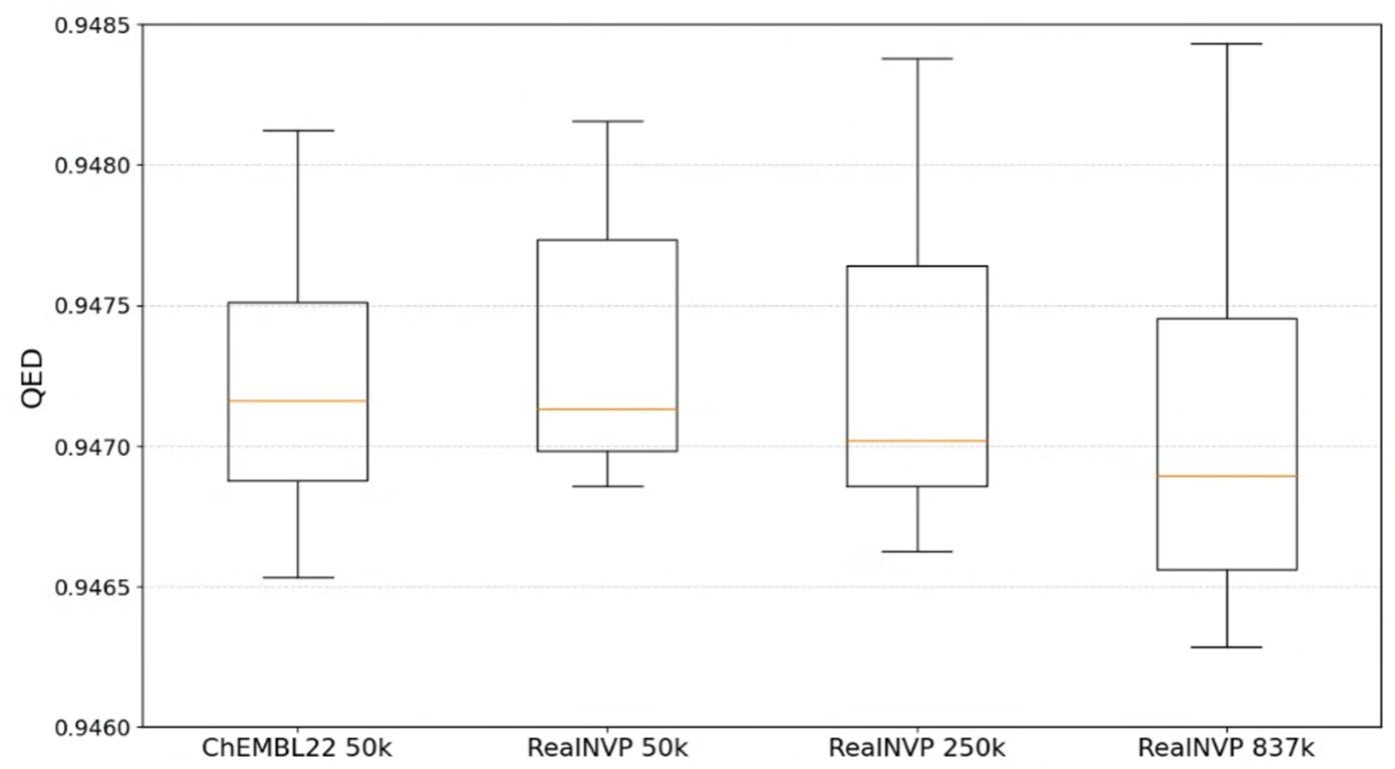
Distributions of QED values for molecules with the highest $0.1\%$ QED score in: 50,000 random samples from ChEMBL22 (training dataset), and the first 50,000, 250,000, and 837,000 generated molecules. These molecules were sampled using Real NVP normalizing flows with VAE encoding defined over one-hot-encoded SMILES. The number of data points visualized from left to right is 50, 50, 250, and 837, respectively. The mean QED values are in yellow

**Fig. 6:**
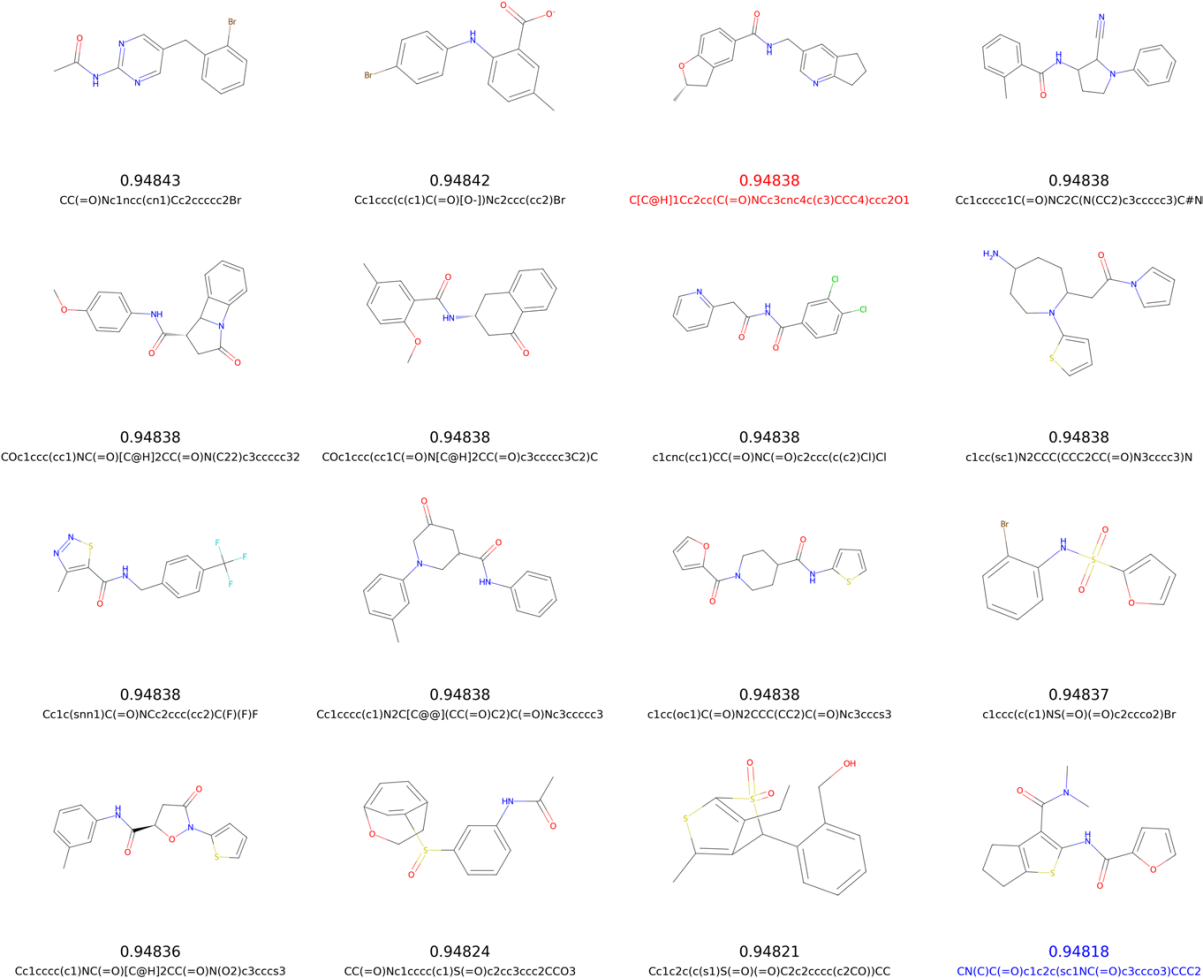
The highest QED scores, the respective molecules, and their SMILES strings in the generated dataset (shown in black) and the ChEMBL dataset (for the 50,000 training dataset and the entire 2.4 million database, shown in blue and red, respectively). 17 generated molecules with the highest similarity index, calculated using Morgan fingerprint, were omitted from this Figure. The mean similarity index for the displayed molecules is 0.26 while the maximum is 0.33

Surprisingly, there was only minor degradation in performance as more than 50,000 samples were generated. This is evident in the distributions of the top $0.1\%$ QED scores and their means for the training and generated datasets, shown in Fig. 5. We did not observe an improvement in the maximum QED score in the previous tests, but it improved with the first 50,000 samples, followed by further smaller improvements in the subsequent samples.

Finally, we analyzed the generated molecules that exhibited higher QED scores than any found in the training dataset. Molecules with similar molecular formulas, identical functional groups, or high structural similarity were excluded. The remaining 14 molecules, along with their QED scores, are presented in Fig. 6. The highest QED score in the current version of ChEMBL35 is 0.94837, which was exceeded by four of the generated molecules. Among these, two differed by the presence of a negatively charged carboxyl group, a bromine atom, or a pyrimidine ring. Although we did not observe any significant degradation in sample quality, it is likely that retraining the VAE on the newly generated molecules could lead to further improvements.

### Generation of novel organofluorine-phosphates using normalizing flows without VAE encoding

In this section, we focus on a specific class of molecules that are important in agriculture, industry, and as chemical warfare agent simulants, organofluorine-phosphates. While numerous publicly available datasets exist for bioactive molecules, none are available for this group of compounds. This is one of the reasons why many of their properties, including toxicity patterns and reactivity, remain poorly understood [62]. There have been efforts to establish empirical guidelines for the assessment of reactivity, and the electronic density on the central phosphorus atom has been identified as a good first approximation [63]. It has been proposed that a higher electronic density at this center enhances the ability of the phosphorus atom to undergo nucleophilic attack, owing to increased electrostatic interactions with the negatively charged hydroxide nucleophile [63]. The electronic density can be approximated by an atomic electronic charge and computed using electronic structure methods based on DFT.

Since there is no comprehensive database of organofluorine-phosphates, training a specialized VAE model for this compound class is not feasible. While one could apply a VAE pre-trained on general bioactive molecules or append the desired functional group to structures from an existing database, such approaches are likely to result in a highly biased training set and low validity rates. The latter arises from the fact that the underlying molecular scaffolds may not readily accommodate the desired functional groups. Furthermore, this application represents a more challenging test case for our approach than simply re-using generated molecules from the previous chapter by appending the organofluorine-phosphate functional group to randomly generated molecules. In such case, one could expect a performance very close to the results presented in the Table 1 (rows “Random”).

Thus, to demonstrate that normalizing flows can be effectively utilized in low-data regimes, we adopted molecular representations that are inherently robust and do not rely on VAEs to achieve high validity rates of generated molecules. In particular, we employed SELFIES and group-SELFIES, both of which are designed to produce syntactically valid molecular strings by construction [34].

**Fig. 7:**
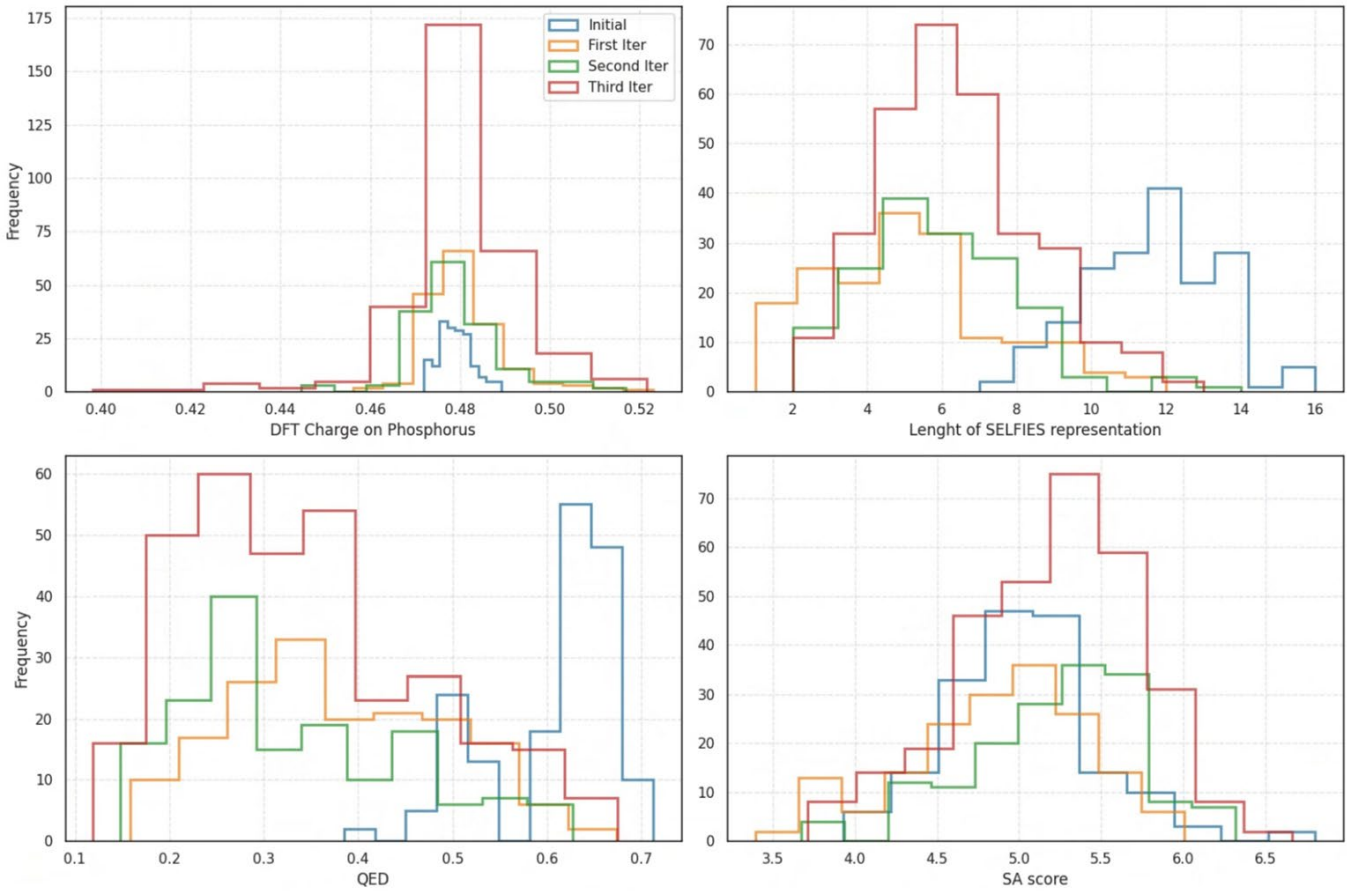
Histograms of Hirshfeld charges on phosphorus atoms, length of SELFIES encoding (exluding the organofluorine-phosphate functional group), QED and SA score for the intial dataset and compared to those from the datasets generated in the first, second and third iterations

**Fig. 8:**
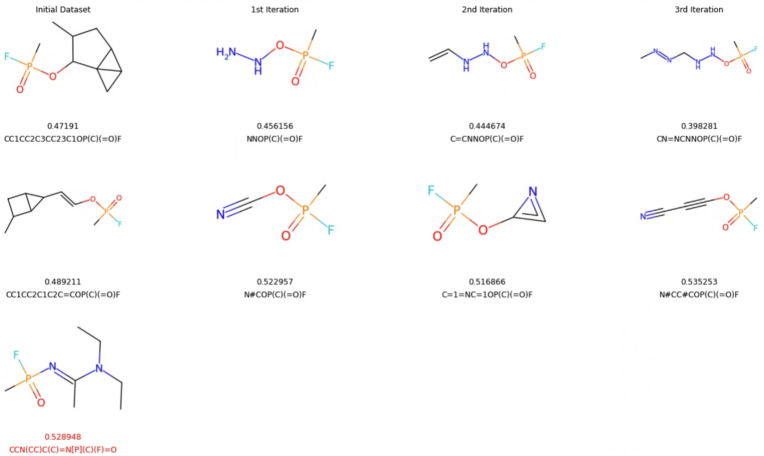
The structures of organofluorine-phosphate molecules with the lowest and highest scores from the initial dataset are compared to those in the first, second and third iterations. For reference, the structure and analysis of the A230 molecule (also known as the Novichok agent) are shown in red at the bottom

We initiated our search with a dataset of only 175 molecular examples published in a previous study that employed recurrent neural networks (RNNs), so we adopted an iterative architecture designed to improve and expand the training dataset over the course of the simulation [64]. The training dataset consists primarily of molecules composed of carbon atom chains and rings, containing only a limited number of functional groups or aromatic systems. The distribution of their phosphorus atomic charges is shown in blue in the top-left panel of Fig. 7. Using this limited dataset, we were not successful in directly generating organofluorine-phosphates using normalizing flows; instead, we trained the model to generate the scaffolds capable of accommodating the attachment of the organofluorine-phosphate functional group, e.g., –OP(C)(=O)F (see Fig. 8 for examples). These scaffolds were subsequently used in the construction of organofluorine-phosphates.

We used conditional Real NVP flows with eight coupling layers and three hidden layers with 128 features each. Note the increase in model complexity compared to the previous application using a VAE, where only four coupling layers and 32 features were sufficient. This increase in complexity was driven by the higher feature dimensionality, which no longer depends solely on the VAE latent space dimension (292), but instead on the maximum length of the SELFIES encoding (equal to 16, the longest SELFIES string in the initial training dataset) and the total number of unique SELFIES tokens (17). After adding a capping empty token to make all the samples of the same length, this resulted in one-hot-encoded molecular representation with a length of 288. This dimension can rapidly increase when longer sequences and/or more tokens are considered to the point when it would no longer be feasible to effective use normalizing flows without application of a feature reduction technique. Finally, since our goal is to improve and expand the training dataset over the course of the simulation and to learn scaffolds rather than complete molecules, we opted to over-train rather than under-train the model in the beginning of the simulation.

We generated new samples in three successive iterations (500, 500, and 1000, respectively). Out of the 2000 generated samples, 61% (1224) were valid and unique scaffolds. This represents a notable decline compared to previously reported SELFIES validity rates, primarily due to the added requirement that the generated scaffolds must be compatible with the attachment of the –OP(C)(=O)F functional group at the end of the string. From these 1224 valid and unique organofluorine-phosphate molecules, we successfully determined 3D coordinates and Hirshfeld atomic charges for 55% (673 molecules) using the DFT method. These structures also exhibited positive normal modes which is indicative of the stability of the optimized geometries. Computational details are provided in Sect. 2.2 and examples of the generated structures can be found in columns 2, 3, and 4 of Fig. 8.

The distributions of charges, SELFIES string lengths, QED scores, and SA scores are shown in Fig. 7 and can be summarized as follows. No significant shift was observed in the distributions of charges on phosphorus atoms (as shown in the top-left panel). The median value remained at approximately 0.48 a.u. across the newly generated molecules over all three iterations. Nevertheless, we successfully produced examples with both higher and lower phosphorus charges than those found in the original dataset (shown in blue), indicating increased diversity. This suggests that the normalizing flow model was able to generate out-of-distribution samples consistently across all three iterative loops, rather than only in the initial iteration, as it was trained on both the original and previously generated data. Thus, we were successful in generating samples outside of the distribution.

After discarding token strings that do not form a valid part of a molecule (which are skipped in the SELFIES translation process), most of the resulting SELFIES strings and the resulting molecules are relatively short (see the top-right panel for the distribution of SELFIES lengths). This leads to a substantial decrease in the QED score, which can be attributed to several factors. First, we restricted the length of the SELFIES strings to match the maximum length in the initial dataset (16 tokens). In practice, we generated shorter sequences that included the capping token, resulting in considerably shorter SELFIES compared to those in the initial dataset. This issue is exacerbated by the SELFIES translation process, which skips tokens that do not form a valid part of a molecule. As a result, the length of the SELFIES is effectively shortened and the generated molecules are smaller.

We believe that using normalizing flows to generate a specific molecular type is not straightforward, as the normalizing flows approach learns molecular structures in a holistic way and does not learn shorter sequences within the molecules. This challenge may be further exacerbated by the large number of branch and ring tokens present in the training data, which complicates the generation of new samples. Additionally, we observed that our protocol was unable to generate molecules featuring aromatic rings. Therefore, a richer molecular representation, a more expressive normalizing flow model, or an expanded training dataset would be necessary to effectively learn such molecular structures. To address some of the deficiencies, we test a novel molecular representation called group-SELFIES next [34].

#### Performance of the group-SELFIES

**Fig. 9:**
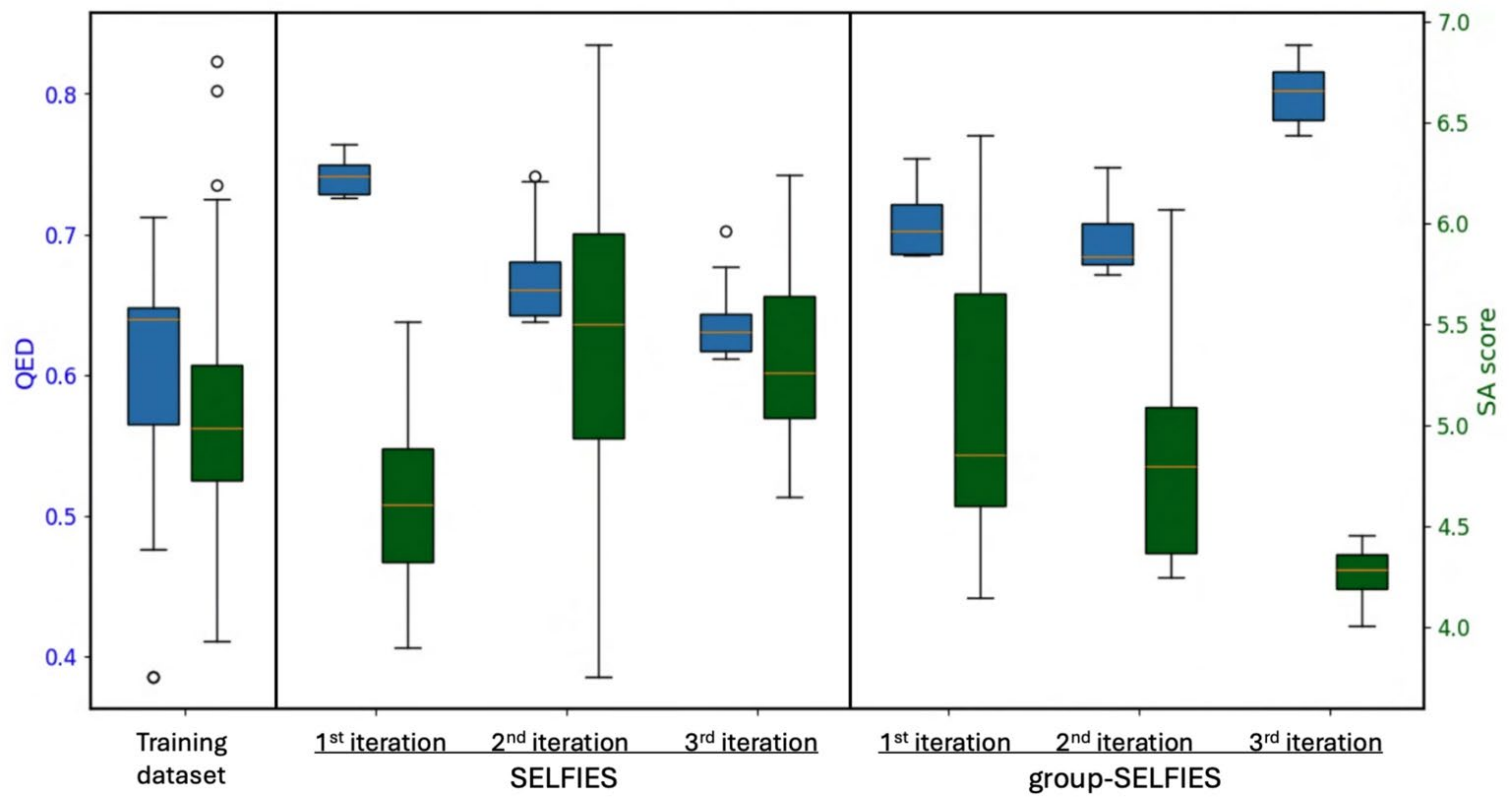
Distributions of QED values for molecules in the training dataset and the top 1% of generated molecules (ranked by QED) across three iterations. Generation was performed using Real NVP normalizing flows with one-hot-encoded SELFIES and group-SELFIES representations

To address several of the limitations observed in the previous section (such as the small molecular size, low QED scores, and the low occurrence of aromatic bonds), we now turn to a novel molecular representation known as group-SELFIES [34]. This approach builds upon standard SELFIES by incorporating an additional step: the definition of molecular fragments. By grouping atoms into chemically meaningful substructures, group-SELFIES offers a more compact and expressive representation, thereby improving the effectiveness of molecular generation. To keep the vocabulary limited while promoting diversity of the generated samples, we extracted 10 aromatic ring fragments from the ZINC250k database using RDKit fragmentation tool. To inspect these fragments, see Fig. 12. Furthermore, to allow the generation of larger molecules, we increased the maximum length of the generated strings from 16 tokens to 30 tokens (Fig. 9).

**Fig. 10:**
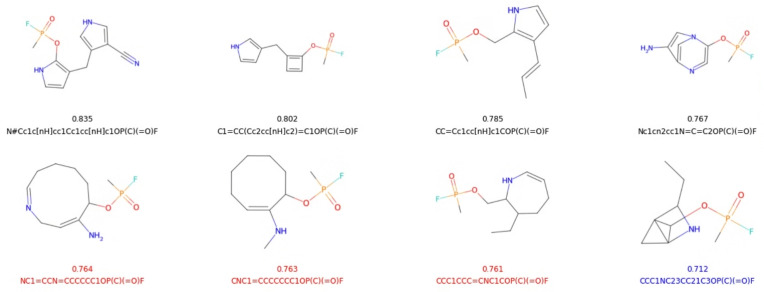
The molecule with the highest QED in the training dataset, molecules generated using one-hot-encoded SELFIES and group-SELFIES are in blue, red, and black, respectively. Their QED scores and SMILES strings are included under the corresponding molecular structures for reference

Table 2 compares the initial dataset with the generated molecules using the two representations, SELFIES and group-SELFIES. The mean QED score, SA score and number of heavy atoms were the same for both models: 0.40, 5, and 11, respectively. The SELFIES had a higher validity rate compared to the group-SELFIES model, resulting in 7329 and 3212 valid and unique molecules. This also resulted in almost twice the number of SELFIES samples with QED higher than any of the molecules in the training dataset: 70 compared to 39 for group-SELFIES (see the top examples in Fig. 10).

Out of the 39 samples, all contained a ZINC250k aromatic ring fragment. While the SELFIES model generated several molecules with aromatic rings, these did not achieve high QED scores. Interestingly, the generated organofluorine-phosphate molecule with the second-highest QED score contains a cyclobutadiene, which has well-known antiaromatic properties. Although sterically bulky substituents or metal ligand binding can stabilize this moiety, it is not expected to be the case here [65]. This serves as a reminder that our molecular representation is not chemistry-aware, and generation under synthetic constraints is one of the possible future directions of this work [66].

Both models were able to generate samples with higher QED score than any in the training dataset. However, group-SELFIES generated samples with a higher maximum QED score of 0.83, compared to 0.76 generated using the SELFIES encoding (see Table 2). Finally, while we observed a decline in the high QED scores in the later iterations using the SELFIES encoding, molecules generated using group-SELFIES continued to improve. This could be due to the small size of the training dataset, which lacks the fragments present in the ZINC250k database. These missing examples are only added as the simulation progresses.

**Table 2:** Test of the group-SELFIES and SELFIES representations in a generative task using Real NVP normalizing flows with 8 layers, each containing 128 neurons

Representation	Generated	QED	SA score	Heavy atoms	Aromatic
	structures	mean/max	mean	mean	rings$^*$
SELFIES	7329	0.40/0.76	5.3	11.6	0$\%$
Group-SELFIES	3212	0.40/0.83	5.7	11.7	8$\%$ (5%)
Initial dataset	175	0.61/0.71	5.0	12.7	0%

$^*$ The percentage in the bracket for group-SELFIES is the occurance of the samples containing any group-SELFIES token in the generated valid string

The training set consisted of 175 organofluorine-phosphate molecules. Three iterations were performed, with each iteration enriching the training set with all the generated examples from the previous iterations

Finally, let us briefly comment on the use of group-SELFIES [34]. This representation offers a promising improvement over traditional molecular encodings, as it allows longer sequences to be compressed by defining chemically meaningful fragments. However, the number of unique tokens grows rapidly with dataset size, as each fragment can have multiple variants depending on how it is connected to the rest of the molecule. This leads to an increasingly large alphabet. As a result, even with a fixed maximum string length, the dimensionality of the one-hot-encoded representation increases, complicating its application in normalizing flows. This limits the model performance and requires more complex model architectures, as normalizing flows rely on relatively simple transformations and often struggle with high-dimensional, complex distributions.

## Conclusions

In this paper, we presented the applications of normalizing flows to molecular design and integrated variational autoencoders with normalizing flows into a comprehensive workflow. We showed that normalizing flows can be effectively combined with one-hot-encoded SMILES representations compressed and transformed using variational autoencoders, as well as directly with the one-hot-encoded SELFIES and group-SELFIES representations. These approaches yield higher validity scores compared to the SMILES representation, as expected.

Additionally, we found that normalizing flows can improve the sampling efficiency of other models, such as variational autoencoders. While the increase in the percentage of valid and unique samples was relatively modest, the mean QED score more than doubled, the maximum QED score increased, and the mean SA score improved by approximately 40%. We identified several novel molecules with QED scores exceeding those found in the 2.4 million-compound ChEMBL database, highlighting the potential of our approach for discovering highly promising drug candidates.

Normalizing flows were capable of learning highly non-Gaussian posterior densities, including those found in discrete chemical space in the molecular design. Furthermore, normalizing flow-based models can be conditioned on additional variables (for instance a costly target metrics such as binding energies or electronic charges) which could be leveraged to develop goal-oriented algorithms, targeted therapies and optimization of complex molecular objectives.

Further work is needed to develop suitable molecular representations that are both sufficiently expressive and efficient in order to leverage normalizing flows. Currently, the field of normalizing flows is mature enough to both support the development of improved sampling strategies for a wide range of models and serve as a promising competitive alternative to methods like Monte Carlo when domain knowledge has a potential to improve sampling. Normalizing flows are particularly well-suited for domains with sparse data such as material design for high-entropy alloys. It remains to be seen whether our results continue to hold in these and other domains, which we plan to explore in our future research.

## Supplementary Information

See the supplementary material for  Figs. 11, 12, 13, 14, 15, 16 and 17 and Table 3.

Supplementary material 1.

## Data Availability

The code and data were made available at https://github.com/nrc-cnrc/VNFlow
