# Supplementary material for: VNFlow: integration of variational autoencoders and normalizing flows for novel molecular design
Source: J Cheminform. 2025 Oct 24;17:161. doi: 10.1186/s13321-025-01104-2 (PMC12553153; doi:10.1186/s13321-025-01104-2)
Supplement: Supplementary file 1 — Supplementary material 1. [file 13321_2025_1104_MOESM1_ESM.pdf]

Supplementary Information for:

VNFlow: Integration of Variational Autoencoders and Normalizing Flows for Novel Molecular Design

Jiří Hostaš, Mohammad S. Ghaemi, Hang Hu, Junan Lin, Anguang Hu, and Hsu Kiang Ooi

September 30, 2025

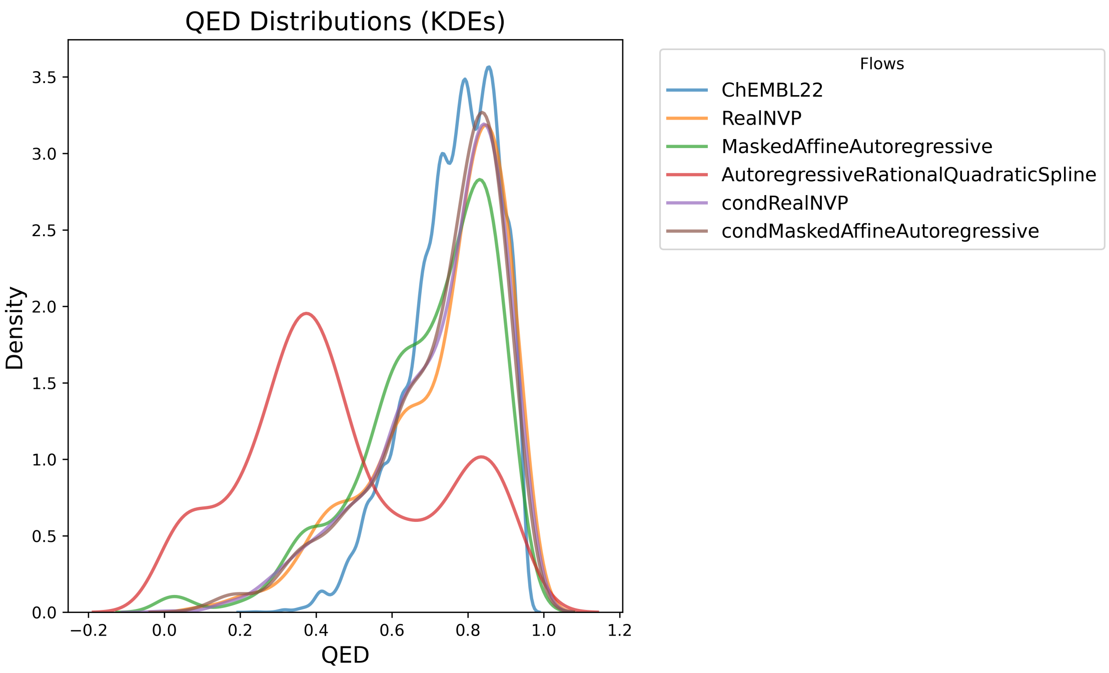

Figure 11: Kernel Density Estimation (KDE) of QED distributions for the starting dataset (50,000 structures from ChEMBL daset) compared with those generated by different normalizing flows. Please note that QED values have a range [0, 1] despite the KDE having non-zero values outside of this range.

Table 3: Hyperparameter search for VAE model trained on one-hot-encoded SELFIES. Decoder architecture was kept fixed while number of convolutional layers used in the encoder was varied.

Model	Convolutional layers and their kernel size	Training Loss	Validation Loss
Chosen model	(9, 9, 9)	17.1	27.4
Setting #2	(9, 9, 9, 9)	39.8	46.4
Setting #3	(12, 12, 12)	17.0	27.8
Setting #4	(12, 12, 12, 12)	27.0	37.1

Note: The progression of training and validation losses of the chosen model is depicted in the Figure 15.

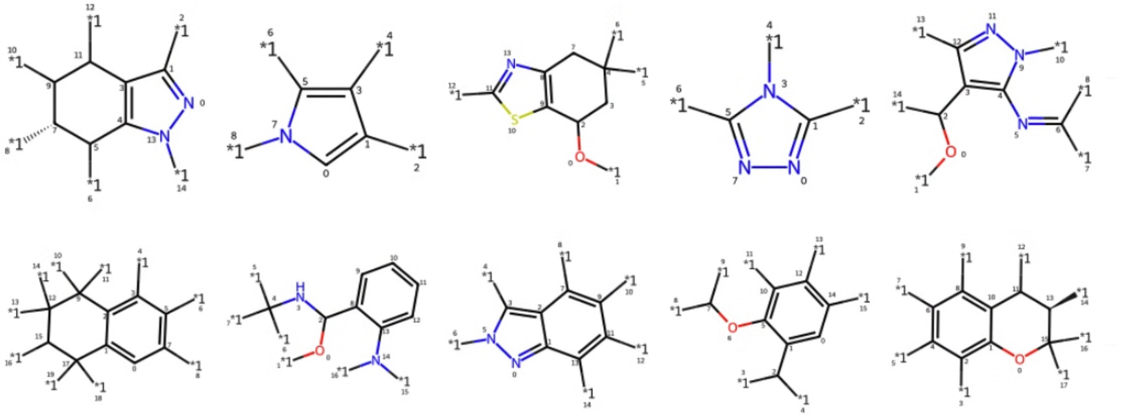

Figure 12: Fragments containing aromatic rings and generated from ZINC250k database.

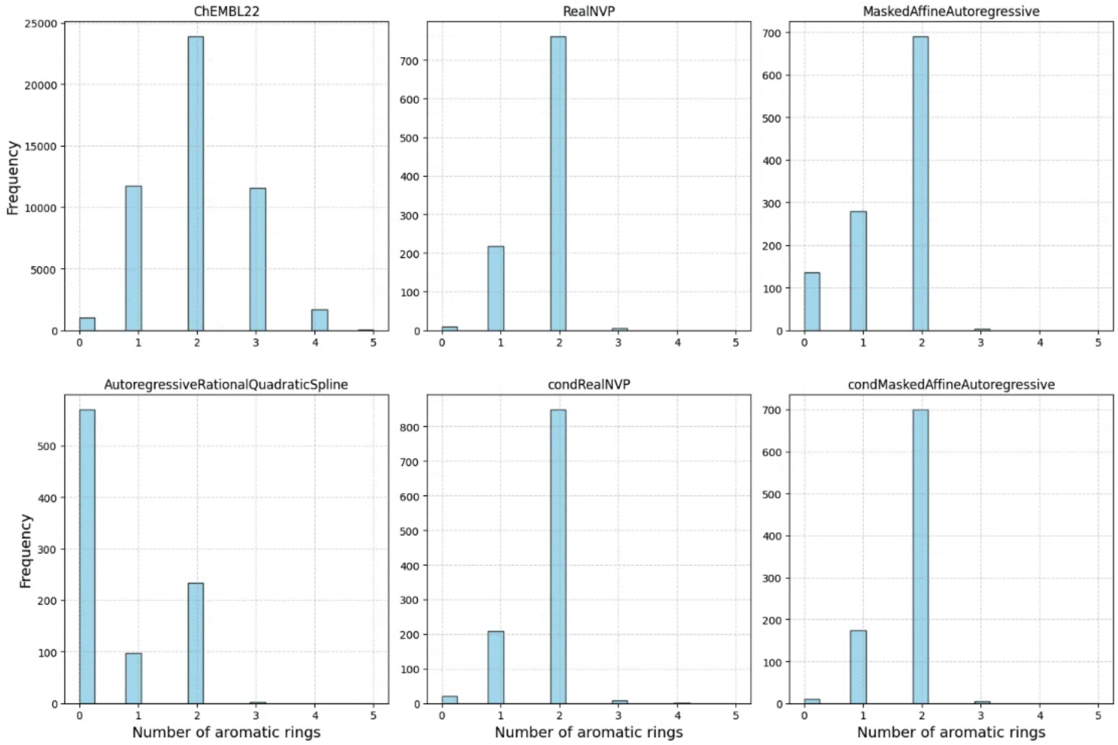

Figure 13: The number of aromatic rings in the starting dataset (50,000 structres from ChEMBL daset) compared with those generated by different normalizing flows. For comparison, the random latent vectors decoded by VAE resulted in 735 non-aromatic molecules, 16 molecules with one aromatic ring and 1 molecule with two.

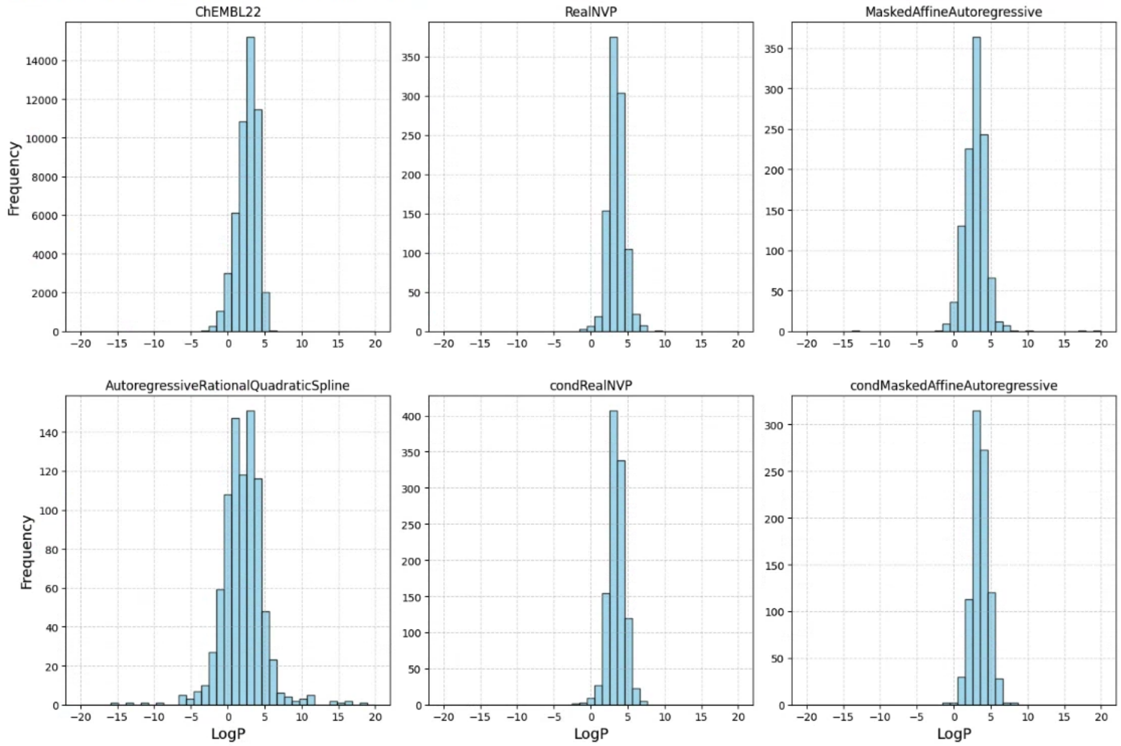

Figure 14: The logP values in the starting dataset (50,000 structres from ChEMBL daset) compared with those generated by different normalizing flows.

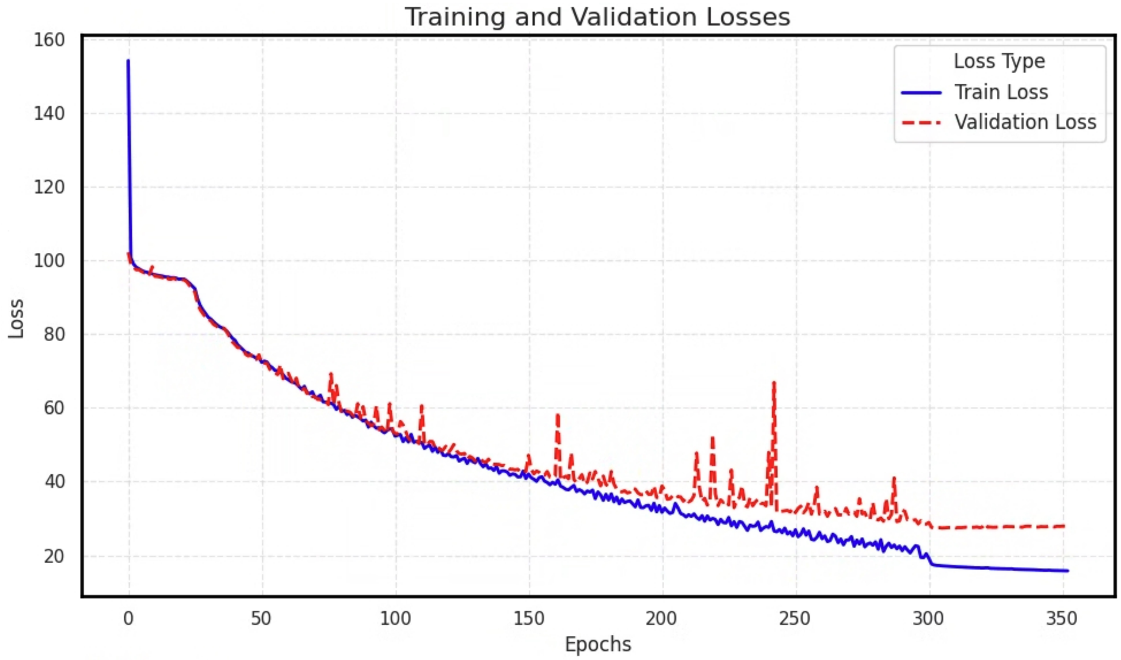

Figure 15: Training and Validation losses during the optimization of VAE model using one-hot-encoded SELFIES as input.

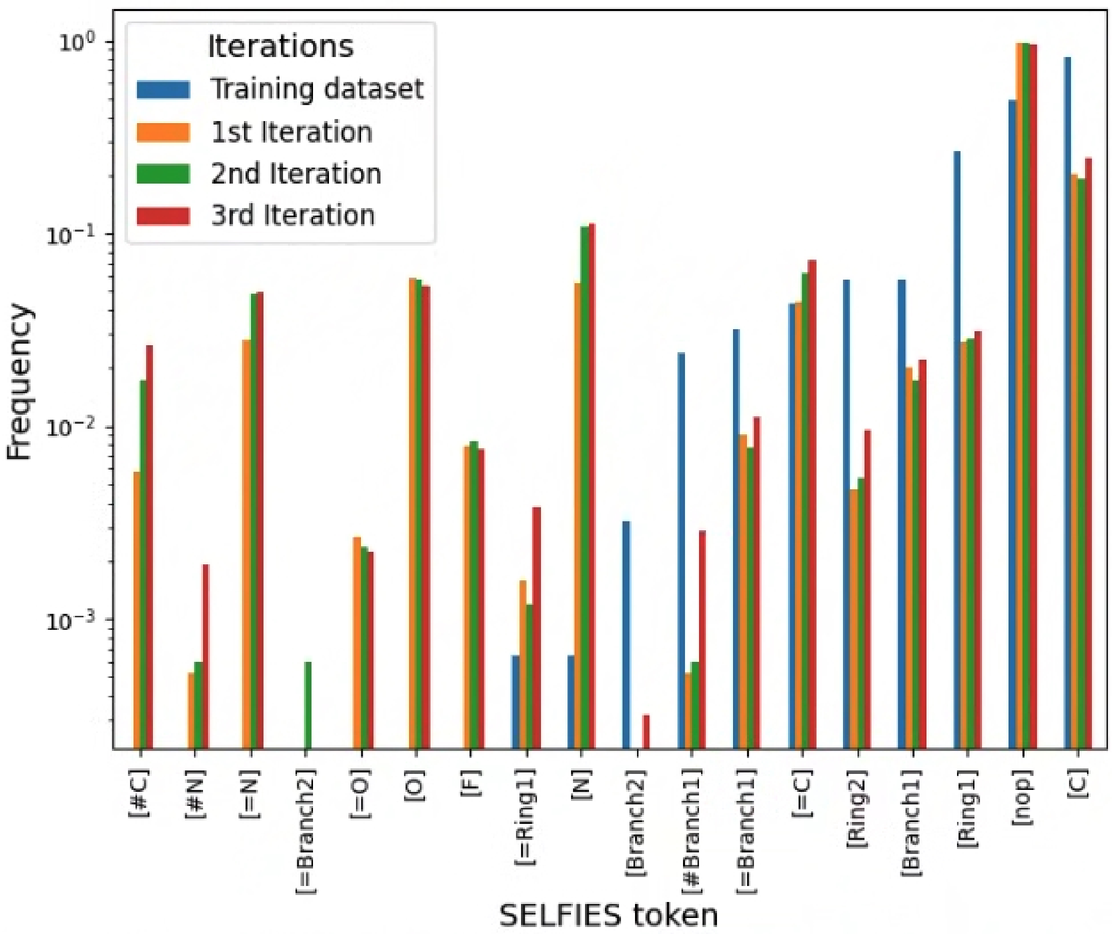

Figure 16: Visualization of the occurrence of SELFIES tokens for the first three iterations (see details in Section 3.2). Tokens are ordered based on their frequency in the training dataset. The frequency, plotted on a logarithmic scale, is calculated as the ratio of valid samples containing each token in the dataset.

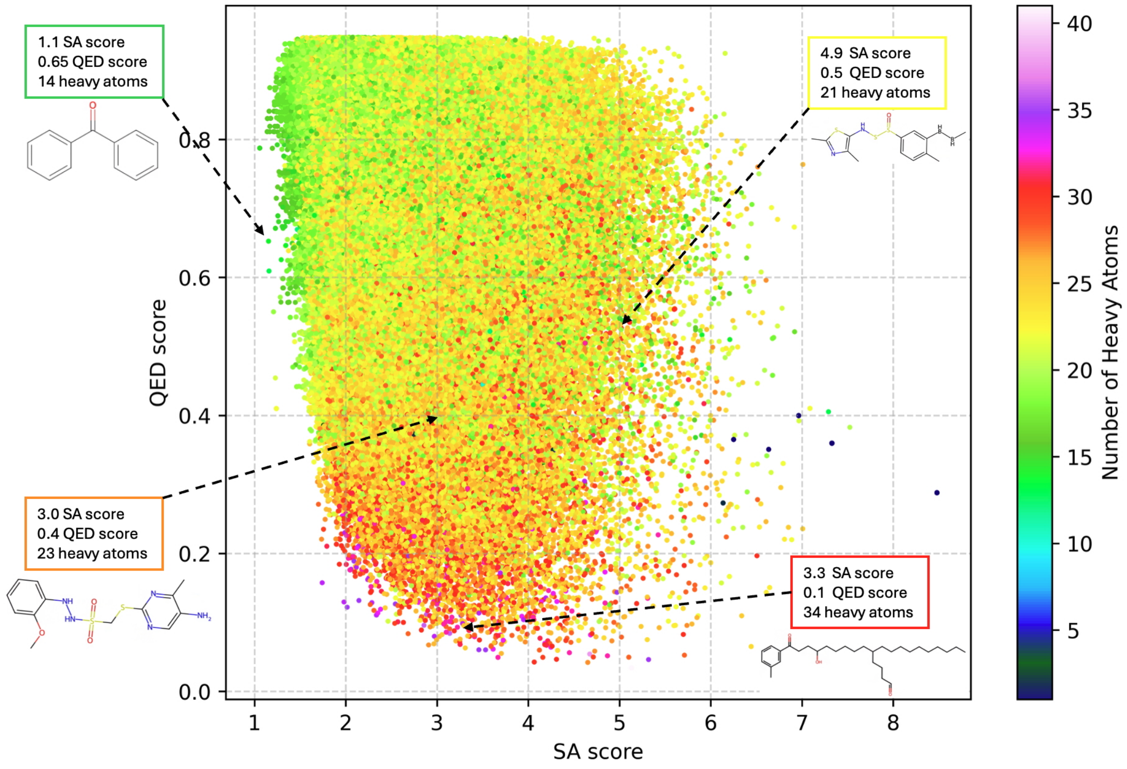

Figure 17: Visualization of 250k generated molecules sampled across chemical space, including four representative molecular examples. Further details are provided in Section 3.1.1, and the samples are available in the GitHub repository.
